# Fast Detection of Phenolic Compounds in Extracts of Easter Pears (*Pyrus communis*) from the Atacama Desert by Ultrahigh-Performance Liquid Chromatography and Mass Spectrometry (UHPLC–Q/Orbitrap/MS/MS)

**DOI:** 10.3390/molecules21010092

**Published:** 2016-01-15

**Authors:** Mario J. Simirgiotis, Cristina Quispe, Jorge Bórquez, Carlos Areche, Beatriz Sepúlveda

**Affiliations:** 1 Laboratorio de Productos Naturales, Departamento de Química, Facultad de Ciencias Básicas, Universidad de Antofagasta, Av. Coloso S-N, Antofagasta 1240000, Chile; jorge.borquez@uantof.cl; 2 Facultad de Ciencias de la Salud, Universidad Arturo Prat, Casilla 121, Iquique 1110939, Chile; elquispe@unap.cl; 3 Departamento de Química, Facultad de Ciencias, Universidad de Chile, Casilla 653, Santiago 7800024, Chile; areche@uchile.cl; 4 Departamento de Ciencias Químicas, Universidad Andres Bello, Campus Viña del Mar, Quillota 980, Viña del Mar 2520000, Chile; bsepulveda@uc.cl

**Keywords:** Chilean pears, Pera de Pascua, phenolics, antioxidant capacity, UHPLC-MS, Orbitrap (OT), proanthocyanidins, flavonoids

## Abstract

A small Chilean variety of pears growing in the town of Toconao, an oasis located at the northeastern edge of the Salar de Atacama, northern Chile, was studied by means of modern PDA and high resolution mass spectral data (UHPLC-PDA-HESI-orbitrap-MS/MS). In addition, the antioxidant features of the fruits were compared with the varieties Packhman’s Triumph and Abate Fetel and correlated with the presence of phenolic compounds. The non-pigmented phenolics were fingerprinted and related to the antioxidant capacities measured by the bleaching of the DPPH radical, the ferric reducing antioxidant power (FRAP), the superoxide anion scavenging activity assay (SA), and total content of phenolics and flavonoids measured by spectroscopic methods. The machine allowed a fast separation of 15 min employing a flow rate of 1 mL per minute and could accurately identify 25 compounds, including several isorhamnetin derivatives and phenolic acids, present in the peel and pulps of this Chilean variety for the first time. The compounds were monitored using a wavelength range of 210–800 nm. The native small Chilean pear showed the highest antioxidant activity measured as the bleaching of the DPPH radical, the ferric reducing antioxidant power and superoxide anion scavenging activity (8.61 ± 0.65 μg/mL, 712.63 ± 12.12 micromols trolox equivalents (μmol/TE)/100 g FW, and 82.89% ± 2.52% at 100 μg/mL, respectively).

## 1. Introduction

**Figure 1:**
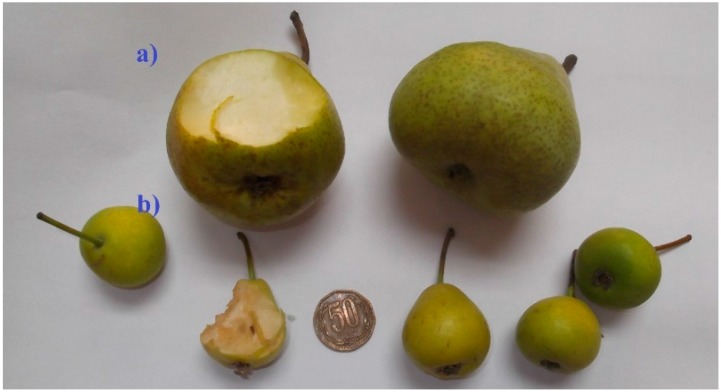
Pictures of (**a**) Packam’s Triumph pears and (**b**) Easter pears from the II region of Chile.

Consumers today are seeking organic natural foodstuffs in order to have a better diet, and this has led to an increase in the daily intake of fruit. Pear (*Pyrus* spp.) fruit is one of the most popular consumed fruits around the world, due to its pleasant taste and nutritional value. This fruit is commonly found in processed products such as baby foods, drinks, marmalades, preserved products and jams. Pears have a substantial amount of carbohydrates, vitamins, minerals and phenolic compounds and present low acidity. There is a great diversity of pear varieties with different appearance in the world due to its widespread consumption and each variety shows a different quantity of nutrients and phenolics, and thus, antioxidant and other beneficial biological activities. The reported compounds in pears comprise arbutin, chlorogenic acid, catechin, quercetin, kaempferol, hydroxycinnamoylmalic acids and their derivatives, procyanidins and triterpenes. In Chile Packam’s Triumph and Beurre Bosc pears comprise more than 60% of Chile’s exports, while Easter Pears (known locally as Pera de Pascua) are small pears produced by local inhabitants in desert valleys in northern Chile, cultivated only for local consumption. The use of liquid chromatography (HPLC, UPLC) with ESI or APCI interphases and mass spectrometers such as quadrupole time of flight (Q-TOF) or quadrupole-electrospray ionization (Q-ESI) analyzers have been used in the last years for metabolic profiling and biological analysis [1,2,3]. The HPLC or UHPLC-MS systems are better than GC-MS since no prior derivatization of samples is necessary [4]. Indeed, quality control of several medicinal plants and drugs plants has also been performed with ESI-MS [5]. HPLC-ESI-MS was used for the analysis of carotenoids [6], anthocyanins [7], phenolic acids [8] and alkaloids [9] in edible fruits and flowers, among other constituents. The Q-Exactive Focus is a recently released hybrid high resolution mass spectrometer with an innovative technology which delivers high resolution MS^/^MS fragments for metabolomics analysis of a variety of species, including toxins, pesticides, antibiotics, peptides and several small organic molecules up to 2000 amu [10,11]. Since we were not able to find HPLC-MS analysis nor phytochemical compounds reported from this Chilean pear variety, and continuing our search for interesting polyphenols in native Chilean plants [12,13,14,15,16] in the present work the polyphenolic fingerprints and phenolic content of edible pears (Figure 1) from the II region of Chile (collected in Toconao, an oasis located at the northeastern edge of the Salar de Atacama) were correlated with the antioxidant capacities measured by the bleaching of the DPPH radical, the ferric reducing antioxidant power (FRAP) and the superoxide anion scavenging activity assay (SA). The compounds were identified for the first time with the help of PDA analysis and high resolution Orbitrap mass spectrometry (HPLC-ESI-Orbitrap-MS) plus comparison with authentic standards. The ethanolic extracts of peel and pulps of the native pears were analyzed and compared with two other Chilean pears, namely Packman’s Triumph and Beurre Bosc pears. In addition, the antioxidant features of the fruits were compared and correlated with the presence of the phenolic compounds. The optimal conditions for the separation were obtained using a linear gradient of only 15 min with a solvent system of 0.1% aqueous formic acid (solvent A) and MS grade acetonitrile 0.1% formic acid (solvent B) with a flow rate of 1.0 mL·min^−1^.

## 2. Results and Discussion

### 2.1. Antioxidant Capacity and Total Phenolics and Flavonoids Contents

In the present study, we assessed the polyphenolic profile of peels and pulps from three pears consumed in Chile, including a small variety locally called Pera de Pascua (Easter Pear) and evaluated their antioxidant capacity as well as the total phenolic and total flavonoid content by spectrophotometric methods.

**Figure 2:**
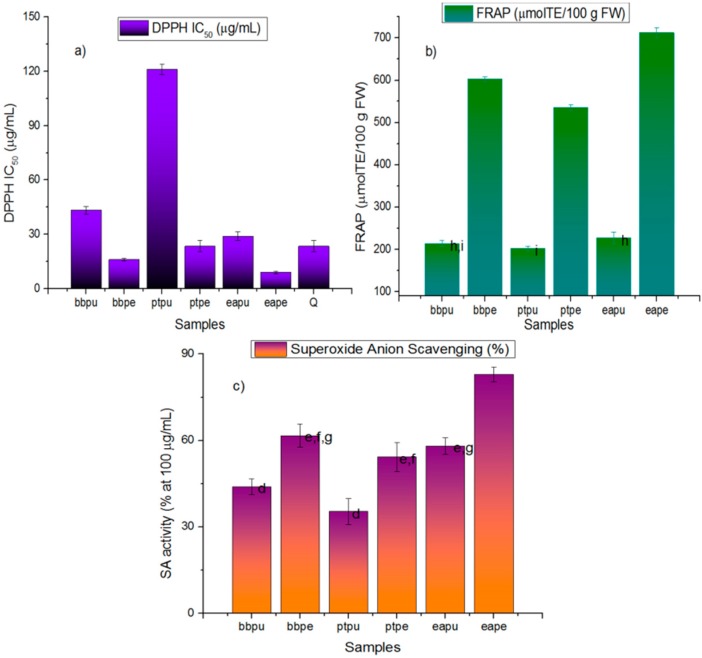

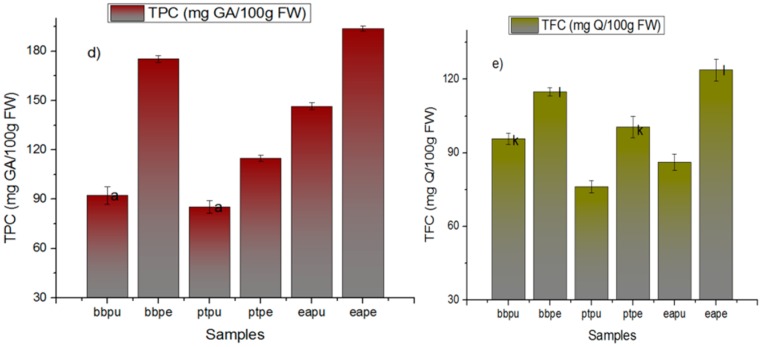
Antioxidant capacities and total phenolic and flavonoid content of three pears growing in Chile. (**a**) DPPH scavenging assay; (**b**) Ferric reducing antioxidant power assay; (**c**) Superoxide anion scavenging assay; (**d**) Total phenolic content and (**e**) Total flavonoid content. (**a**) Expressed as IC_50_ in μg/mL for extracts and compounds. Q: Quercetin; (**b**) Expressed as μM Trolox equivalents/g fresh weight; (**c**) Expressed in percentage scavenging of superoxide anion at 100 μg/mL; (**d**) Expressed as mg gallic acid/100 g fresh weight; (**e**) Expressed as mg quercetin/100 g fresh weight. Bars marked with same letters are not significantly different (at *p* < 0.05). bbpu: Beurre Bosc pear pulp, bbpe: Beurre Bosc pear peel, ptpu: Packam’s triumph pear pulp, ptpe: Packam’s triumph pear peel, eapu: Easter pear pulp, eape: Easter pear peel.

The fruits were purchased from a local market in Antofagasta and the peels and pulps were separately collected. The peels and pulps were extracted with acidified methanol and the resulting extracts were processed by solid phase extraction. Three antioxidant assays were employed for this study. The order of the antioxidant activity measured by the bleaching of the DPPH radical and the ferric reducing antioxidant power (FRAP) showed by the six fruit materials under study were Easter Pear peel > Beurre Bosc peel > Packam’s Triumph peel > Easter Pear pulp > Beurre Bosc pulp > Packam’s Triumph pulp. Easter pear peel showed more DPPH scavenging activity than the standard antioxidant quercetin. A similar trend was observed for superoxide anion scavenging activity (SA, Figure 2). Total phenolic contents were Easter Pear peel > Beurre Bosc peel > Easter Pear pulp > Packam’s Triumph peel > Beurre Bosc pulp > Packam’s Triumph pulp, while total flavonoid contents showed some difference with TPC and were Easter Pear peel > Beurre Bosc peel > Packam’s Triumph peel > Beurre Bosc pulp > Easter Pear pulp > Packam’s Triumph pulp. The problem with the Folin−Ciocalteu method is that this assay frequently overestimates the real phenolics content, as the reagent reacts not only with phenolic compounds, but also with some other antioxidants, such as proteins and some inorganic ions. For instance, the wide variation for phenolics content in several eggplant materials has been matched by high values for variation in antioxidant activity and also variation in chlorogenic acid contents [17]. However, taking into consideration the good correlation between assays, in this study the total phenolics reported for the three varieties from Chile was four times higher to that reported for the Hongpi variety from China [18] and almost three times of the *Pyrus communis* varieties Nakh and Nashpati from Pakistan (around 60 and 33 mg GAE 100 g fresh weight, taking into account 90% of loss of water) [19] and were also almost double to that reported for peel and pulp of Bartlett and Starkrimson pears (around 120/140 and 100/85 mg GAE 100 g fresh weight for peels and 20/7 and 10/5 for pulps) [20], while the total flavonoids showed a slightly different trend (Figure 2) the TFC of Easter pear peel were double (although the values were reported in catechin equivalents not in QE) to that reported from two pear varieties from Pakistan [19] and several fruit peels [12,14] including native Pica mangoes from Chile [21] but lower than Pica lemons [16].

### 2.2. MS-PDA Identification of Phenolic Acids in Pear Fruits from Northern Chile

**Figure 3:**
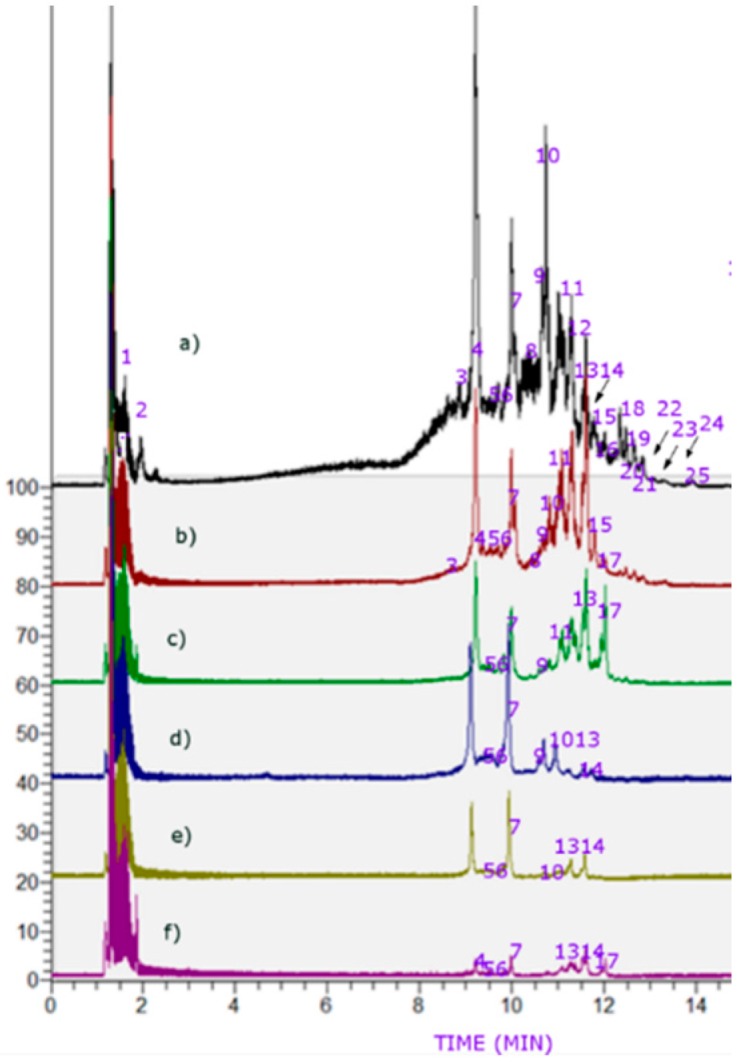
UHPLC-TIC (ultra HPLC total ion current) chromatograms of (**a**) Easter Pear peel (**b**) Beurre Bosc peel (**c**) Beurre Bosc pulp (**d**) Packam’s Triumph pulp (**e**) Packam’s Triumph peel and (**f**) Easter Pear pulp.

We couldn’t avoid some degradation of the compounds, evidenced by some slope in the UHPLC TIC chromatograms of the Easter Pear (Figure 3), even though the extractions were performed under mild conditions (concentrated *in vacuo* below 40 °C and in the dark). However, several phenolic acids were detected in the pear fruits and identified using ultra HPLC with TIC and UV-visible data (PDA, Figure 3, Table 1 and Orbitrap electrospray mass spectrometry (OT-ESI-MS, Table 1). Several common compounds including proanthocyanidins, phenolic acids and flavonoids were already reported in pears [18,22,23] and were identified accurately in the present study by full MS and the fragmentation provided by the HCD cell. Peaks 1–16 and 18–25 were detected in Easter Pear peel, Peaks 1–18, 20–23 in Easter Pear pulp, peaks 1, 2, 4–7, 9, 11–13 and 17 in Beurre Bosc peel, peaks 1–7, 9–11, 13 and 14 in Beurre Bosc pulp, peaks 1, 4–7, 10, 13 and 14 in Packam’s Triumph peel and Peaks 1, 2, 4–7, 10, 13, 14 and 17 in Packam’s Triumph pulp. Below is a detailed explanation of the characterization.

**Table 1:** Identification of Phenolic Compounds in Chilean pears by LC-PDA-HR-OT-ESI-MS Data.

Peak #	Uv Max	Tentative Identification	Molecular Formula	Retention Time	Theoretical Mass	Measured Mass	Accuracy (Dppm)	Other Ions
1	325	caffeoyl-glucose	C_15_H_17_O_9_^−^	1.79	341.0878	341.0872	1.75	191.0555 (quinic acid C_7_H_11_O_6_^−^)
2	330	Unknown quinic acid derivative		1.82		426.9632	−0.5079	191.0193 (quinic acid C_7_H_11_O_6_^−^)
3	236, 326	3-*O*-caffeoylquinic acid (3-CQA) *	C_16_H_17_O_9_^−^	8.63	353.0878	353.0878	0	707.1827 [2M − H]^−^ 191.0556 (quinic acid C_7_H_11_O_6_^−^)
4	236, 329	4-*O*-caffeoylquinic acid (4-CQA)	C_16_H_17_O_9_^−^	9.12	353.0878	353.0876	0.566	191.0555 (quinic acid C_7_H_11_O_6_^−^) (a)
5	280	(+) catechin *	C_15_H_13_O_6_^−^	9.14	289.07176	289.0715	2.7937	
6	279	B-type procyanidin dimer	C_30_H_25_O_12_^−^	9.16	577.13515	577.1349	1.4471	289.0713 (monomer)
7	280	Gallocatechin-3-*O*-glucose	C_21_H_21_O_13_^−^	9.64	481.0987	481.0985	1.6903	
8	236, 329	5-*O*-caffeoylquinic acid (5-CQA) *	C_16_H_17_O_9_^−^	9.87	353.0878	353.0876	0.566	707.1826 [2M − H]^−^ 191.0556 (quinic acid C_7_H_12_O_6_) (b)
9	280	B-type procyanidin dimer monohexose	C_36_H_36_O_27_^−^	10.59	740.6608	739.1668	0.49	
10	240, 325	Feruloyl quinic acid (3-FQA)	C_17_H_19_O_9_^−^	10.65	367.1034	367.1028	1.2398	(c)
11	254, 354	Rutin *	C_27_H_29_O_16_^−^	10.77	609.1461	609.1455		301.0344 (quercetin C_15_H_9_O_7_^−^) (d)
12	254, 354	Quercetin-3-*O*-glucose *	C_21_H_19_O_12_^−^	10.97	463.0882	463.0876	1.1091	301.0353 (Quercetin C_15_H_9_O_7_^−^) (e)
13	254, 354	Isorhamnetin-3-*O*-2′ rhamnosyl)glucose	C_28_H_31_O_16_^−^	11.22	623.1618	623.1617	−0.5079	315.0510 (Isorhamnetin C_16_H_11_O_7_^−^) (f)
14	279	A-type procyanidin dimer	C_30_H_23_O_12_^−^	11.24	575.11950	575.1191	1.1863	
15		*p*-coumaroyl malate	C_13_H_11_O_7_^−^	11.26	279,0510	279.0507	2.8349	
16	254, 354	Isorhamnetin-3-*O*-galactose	C_22_H_21_O_12_^−^	11.49	477.1038	477.1031	0.7041	(g)
17	236, 329	di-*O*-caffeoylquinic acid isomer (di-CQA)	C_25_H_23_O_12_^−^	11.56	515.1195	515.1189	0.9590	
18	254, 354	Isorhamnetin-3-*O*-glucose *	C_22_H_21_O_12_^−^	11.57	477.1038	477.1034	1.2798	
19	236, 329	di-*O*-caffeoylquinic acid isomer (di-CQA)	C_25_H_23_O_12_^−^	11.74	515.1195	515.1191	1.4329	
20	254, 354	isorhamnetin-3-*O*-(6′-acetyl)-glucoside	C_24_H_23_O_13_^−^	11.90	519.1144	519.1138	0.9891	
21	254, 354	isorhamnetin-3-*O*-(6′-acetyl)-galactoside	C_24_H_23_O_13_^−^	11.97	519.1144	519.1141	1.4594	(h)
22	254, 350	3-acetyl-3,5,4′tryhydroxy-7methoxy-flavone	C_18_H_13_O_7_^−^	12.43	341.0667	341.0670	−0.879	
23		A-type procyanidin dimer	C_30_H_23_O_12_^−^	12.60	575.11950	575.1188	0.7528	
24	240, 312	3-*p*-Coumaroylquinic acid	C_18_H_9_O_7_^−^	13.29	337.09289	337.0356	3.9608	
25	254, 347	Kaempferol-3-*O*-glucose	C_21_H_19_O_11_^−^	13.31	447.0721	447.0724	0.671	

* Identified by spiking experiments with authentic compounds. (a) to (h) please see below the full MS^n^ spectra. #: number.

#### 2.2.1. Phenolic Acids

**Figure 4:**
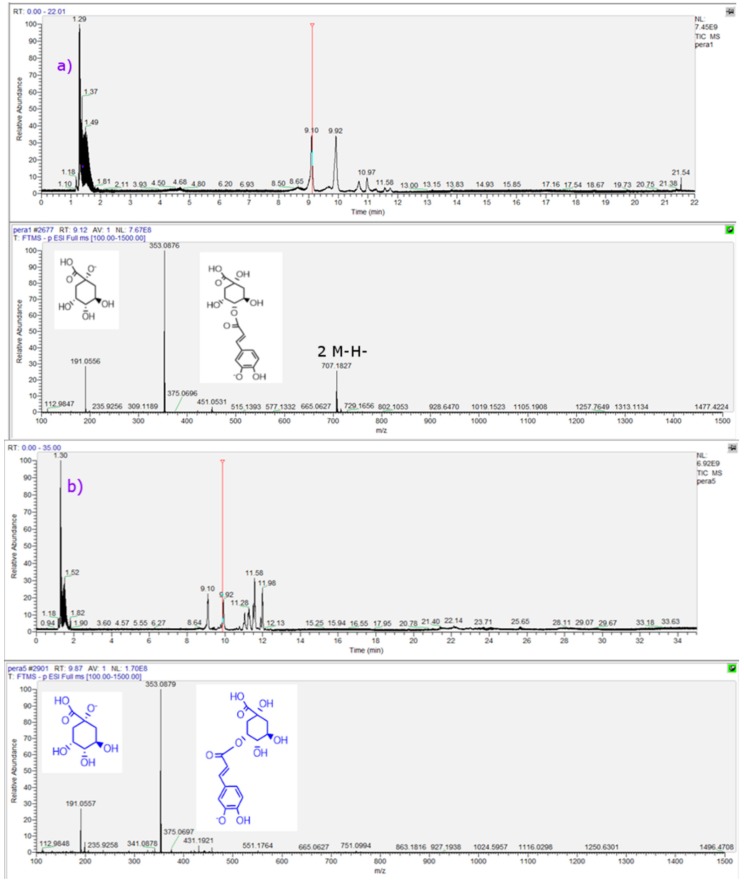

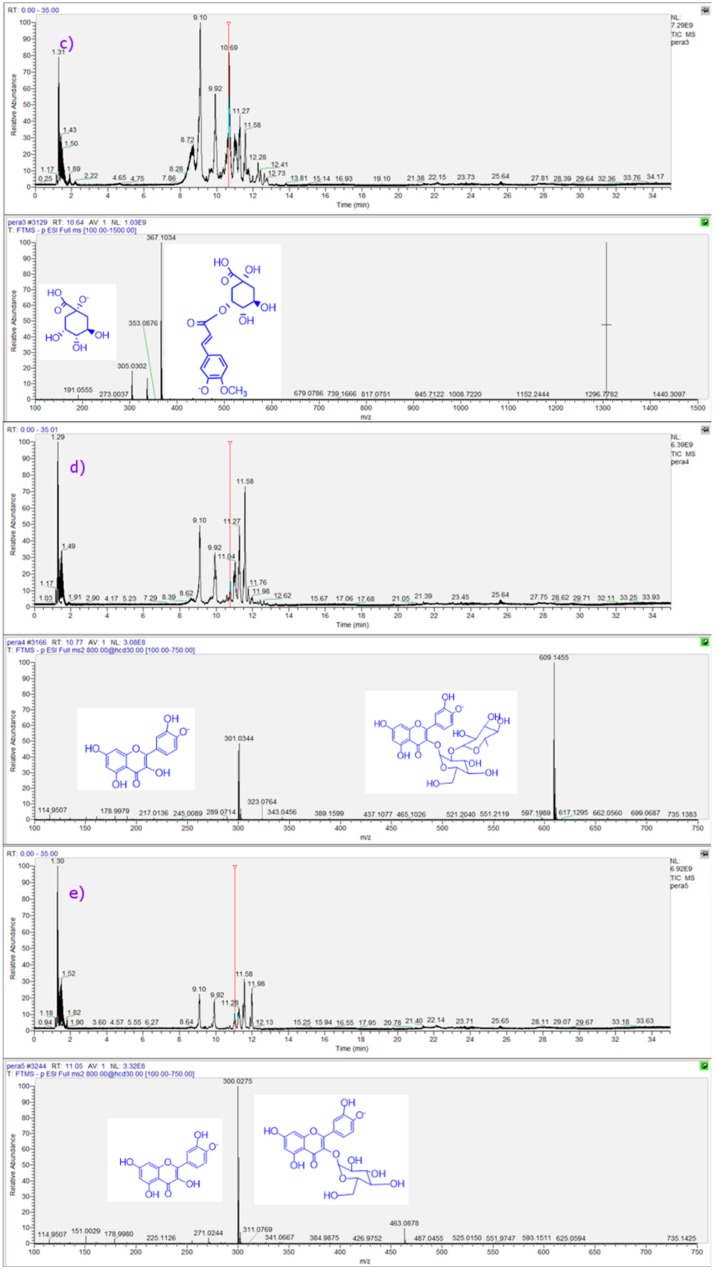

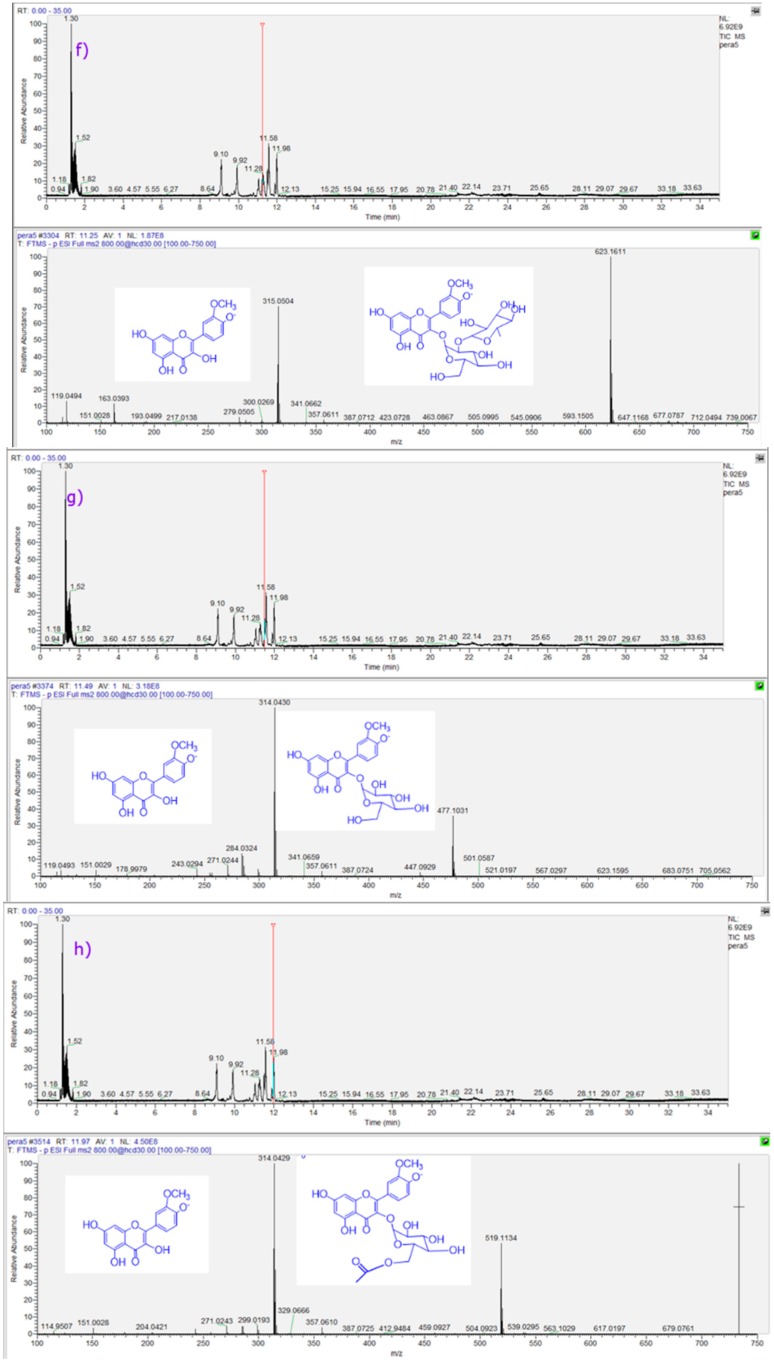
UHPLC-TIC (ultra HPLC total ion current) chromatograms and full scan OT-MS^n^ spectra of some representative compounds identified in three pear fruits cultivated in Chile. (**a**) peak 4; (**b**) peak 8; (**c**) peak 10; (**d**) peak 11; (**e**) peak 12; (**f**) peak 13; (**g**) peak 16; and (**h**) peak 21. Peak numbers refer to those indicated in Table 1.

Three chlorogenic acids (C_16_H_18_O_9_ Figure 4a,b) were identified. The isomers detected include 4-*O*-caffeoylquinic acid (cryptochlorogenic acid or 4-CQA, peak 4), 5-*O*-caffeoylquinic acid (neochlorogenic acid or 5-CQA, peak 4) and 3-*O*-caffeoylquinic acid (chlorogenic acid or 3-CQA, peak 8) [23]. Another peak could be tentatively identified as 3-*O*-feruloylquinic acid (or 3-FQA, peak 10, Figure 4c), all of them producing a quinic acid MS^2^ ion at around *m*/*z*: 191.0556 (quinic acid C_7_H_11_O_6_^−^) and the CGA all produced also a [2M − H]^−^ adduct ion at around *m*/*z*: 707 [24]. They were also identified according to their UV spectra (λ_max_ at 314–330 nm). Peaks 17 and 19 with ions at *m*/*z*: 515.1189 and 515.1191 were identified as di-CQA isomers according to the formula C_25_H_23_O_12_^−^ [25]. Peak 1 was identified as caffeoyl glucoside (341.0872, C_15_H_17_O_9_^−^), while peak 15 as *p*-coumaroyl malate (ion at *m*/*z*: 279.0507, C_13_H_11_O_7_^−^). Peak 24 with a pseudomolecular ion at *m*/*z*: 337.0356 was identified as 3-*p*-coumaroylquinic acid (C_18_H_9_O_7_^−^) [25].

#### 2.2.2. Flavonoids

Isorhamnetin (C_16_H_11_O_7_^−^ λ_max_ 254 354 nm) [13] derivatives were previously reported to occur in pears [26]. Peak 13 with an [M − H]^−^ ion at *m*/*z*: 623.1617 (Figure 4f) corresponding to a molecular formula C_28_H_31_O_16_^−^ producing a MS^2^ ion at *m*/*z*: 315.0510 (isorhamnetin C_16_H_11_O_7_^−^) was tentatively identified as isorhamnetin-3-*O*-(2′-rhamnosyl)glucoside. In the same manner peak 12 with a [M − H]^−^ ion at *m*/*z*: 463,0876 was identified as quercetin-3-*O*-glucoside (C_21_H_19_O_12_^−^) (Figure 4e) [27] which produced a MS^2^ ion at *m*/*z*: 301,0353 (quercetin C_15_H_9_O_7_^−^). Peak 16 with an ion at *m*/*z*: 477.1031 was identified as isorhamnetin-3-*O*-galactoside (C_22_H_21_O_12_^−^) (Figure 4g) while the isomeric peak 18 with a ion at *m*/*z*: 477.1034 was identified as isorhamnetin-3-*O*-glucoside (C_22_H_21_O_12_^−^) [13]. These compounds were identified by spiking experiments with authentic standards. Three isomers with a molecular formula C_24_H_23_O_13_^−^ were also identified as isorhamnetin acylated hexosides (peaks 20 and 21) [23] such as an isorhamnetin-3-*O*-(6′-acetyl)-glucoside and an isorhamnetin-Q-3-*O*-(6’-acetyl)-galactoside (Figure 4h), because those sugars (glucose and galactose) and acyl positions are the most commonly occurring ones in flavonols [28] and peak 11 was identified as rutin (C_27_H_29_O_16_^−^) [24], while peak 25 was identified as kaempferol-3-*O*-glucoside (C_21_H_19_O_11_^−^, pseudomolecular ion at 447.0724) [23].

#### 2.2.3. Procyanidins

Peak 7 with an ion at 481.0987 was identified as gallocatechin-3-*O*-glucoside (C_21_H_21_O_13_^−^), while peak 5 was the monomer (+) catechin (C_15_H_13_O_6_^−^) (catechin, diagnostic fragments at *m*/*z* 245, 205 and 179 [27,29]. Peaks 14 and 23 with pseudomolecular ions at *m*/*z*: 575.1191 and 575.1195, respectively, were identified as A-type procyanidin dimer isomers [30] while peak 6 with a molecular anion at *m*/*z*: 577.1351 was a B-type procyanidin dimer (MS^2^ C_24_H_15_O_9_^−^, monomer), and the related compound assigned to peak 9 with an ion at *m*/*z*: 739.1668 was identified as a B-type procyanidin dimer monohexoside [23,31].

## 3. Experimental Section

### 3.1. Chemicals and Plant Material

Folin-Ciocalteu phenol reagent (2 N), reagent grade Na_2_CO_3_, AlCl_3_, HCl, FeCl_3,_ NaNO_2,_ NaOH, quercetin, trichloroacetic acid, sodium acetate, HPLC-grade water, lichrosolv HPLC-grade acetonitrile, and MeOH, reagent grade MeOH and formic acid were obtained from Merck (Darmstadt, Germany). Rutin, 3-*O*- and 5-*O*-caffeoylquinic acids, quercetin-3-*O*-glucose, isorhamnetin-3-*O*-glucose, (+) catechin, quercetin, isorhamnetin, (all standards with purity higher than 95% by HPLC) were purchased either from Sigma Aldrich (St. Louis, MO, USA), ChromaDex (Santa Ana, CA, USA), Extrasynthèse (Genay, France) or Wuxi Apptec Co. Ltd. (Shanghai, China). Gallic acid, 2,4,6-tri(2-pyridyl)-s-triazine (TPTZ), Trolox, *tert*-butylhydroperoxide, nitroblue tetrazolium, xanthine oxidase and DPPH (1,1-diphenyl-2-picrylhydrazyl radical) were purchased from Sigma-Aldrich Chemical Co. Pear fruits were purchased at La Vega de Antofagasta fruit market in December 2014. Fruit samples were deposited at the Laboratorio de Productos Naturales, Universidad de Antofagasta, Antofagasta, Chile, with the numbers EApear-122314, BBpear-122314 and PTpear-122314.

### 3.2. Sample Preparation

Fresh fruits were carefully washed, separately homogenized in a blender and freeze-dried (Freezone Freeze dry system plus 2.5 L, Labconco Corporation, Kansas City, MO, USA). Ten grams of the lyophilized fruits were finally pulverized in a mortar, defatted thrice with 100 mL of *n*-hexane and then extracted with 100 mL of 0.1% HCl in MeOH in the dark in an ultrasonic bath for one hour each time. The extracts were combined, filtered and evaporated *in vacuo* in the dark (40 °C). The extracts were suspended in 20 mL ultrapure water and loaded onto an XAD-7 (100 g) column. The column was rinsed with water (100 mL) and phenolic compounds were eluted with 100 mL of MeOH acidified with 0.1% HCl. The solutions were combined and evaporated to dryness under reduced pressure (40 °C).

### 3.3. Instruments

A Thermo Scientific Dionex Ultimate 3000 UHPLC system equipped with a quaternary Series RS pump and a Thermo Scientific Dionex Ultimate 3000 Series TCC-3000RS column compartment with a Thermo Fisher Scientific Ultimate 3000 Series WPS-3000RS autosampler and a rapid separations PDA detector controlled by Chromeleon 7.2 Software (Thermo Fisher Scientific, Waltham, MA, USA and Dionex Softron GmbH subsidiary of Thermo Fisher Scientific, Bremen, Germany) hyphenated with a Thermo high resolution Q Exactive focus mass spectrometer were used for analysis. This configuration combines the rapid separation of the UHPLC with PDA detector with flow rates up to 2 mL per minute, zero dead volume, the high resolving power performance of the orbital trap (Orbitrap), and selectivity of a quadrupole, (reaching resolutions of up to 70,000 FWHM at *m*/*z* 200), and the outstanding diagnostic power possible using an HCD cell. The chromatographic system was coupled to the MS with a Heated Electrospray Ionization Source II (HESI II). Nitrogen (purity >99.999%) obtained from a Genius NM32LA nitrogen generator (Peak Scientific, Billerica, MA, USA) was employed as both the collision and damping gas. Mass calibration for Orbitrap was performed once a week, in both negative and positive modes, to ensure a working mass accuracy lowers than or equal to 5 ppm. Caffeine, and *n*-butylamine (Sigma Aldrich) were the calibration standards for positive ions and buspirone hydrochloride, sodium dodecyl sulfate, and taurocholic acid sodium salt (Sigma Aldrich) were used to calibrate the mass spectrometer. These compounds were dissolved in a mixture of acetic acid, acetonitrile, water and methanol (Merck) and were infused using a Chemyx Fusion 100 syringe pump (Thermo Fisher Scientific, Germany). XCalibur 2.3 software (Thermo Fisher Scientific, Germany) and Trace Finder 3.2 (Thermo Fisher Scientific, San José, CA, USA) were used for UHPLC control and data processing, respectively. Q Exactive 2.0 SP 2 from Thermo Fisher Scientific was used to control the mass spectrometer.

### 3.4. LC Parameters

A portion of each extract (5 mg) obtained as explained above was dissolved in 1% formic acid in MeOH (5 mL), filtered through a 0.45 μm micropore membrane (PTFE, Waters, Milford, MA, USA) before use and was injected into the UHPLC-PDA and ESI-Orbitrap-MS equipment. Liquid chromatography was performed using an UHPLC C18 column (Acclaim, 150 mm × 4.6 mm ID, 5 μm, Restek Corporation, Bellefonte PA, USA) operated at 25 °C. The detection wavelengths were 254, 280, 320 and 440 nm, and PDA signal was recorded from 200 to 800 nm for peak characterization. Mobile phases were 1% formic aqueous solution (A) and acetonitrile (B). The gradient program (time (min), % B) was: (0.00, 5); (5.00, 5); (10.00, 30); (15.00, 30); (20.00, 70); (25.00, 70); (35.00, 5) and 12 min for column equilibration before each injection. The flow rate was 1.00 mL·min^−1^, and the injection volume was 10 μL. Standards and extracts dissolved in methanol were kept at 10 °C during storage in the autosampler.

### 3.5. MS Parameters

The HESI parameters were optimized as follows: sheath gas flow rate 75 units; aux. gas unit flow rate 20; capillary temperature 400 °C; aux gas heater temperature 500 °C; spray voltage 2500 V (for ESI^−^); and S lens RF level 30. Full scan data in both positive and negative was acquired at a resolving power of 70,000 FWHM (full width half maximum) at *m*/*z* 200. For the compounds of interest, a scan range of *m*/*z* 100–1000 was chosen; the automatic gain control (AGC) was set at 3E6 and the injection time was set to 200 ms. Scan-rate was set at 2 scans·s^−1^. External calibration was performed using a calibration solution in positive and negative modes before each sample series. In addition to the full scan acquisition method, for confirmations purposes, a targeted MS/MS analysis was performed using the mass inclusion list and expected retention times of the target analytes, with a 30 s time window, with the Orbitrap spectrometer operating both in positive and negative mode at 17,500 FWHM (*m*/*z* 200). The AGC target was set to 2E5, with the maximum injection time of 20 ms. The precursor ions are filtered by the quadrupole which operates at an isolation window of *m*/*z* 2. The fore vacuum, high vacuum and ultrahigh vacuum were maintained at approximately 2 mbar, from 105 and below 1010 mbar, respectively. Collision energy (HCD cell) was operated at 30 kv. Detection was based on calculated exact mass and on retention time of target compounds, presented in Table 1. The mass tolerance window was set to 5 ppm for the two analysis modes.

#### 3.5.1. Ferric Reducing Antioxidant Power

The determination of ferric reducing antioxidant power or ferric reducing ability (FRAP assay) of the extracts was performed as described by [32,33] with some modifications. The stock solutions prepared were 300 mM acetate buffer pH 3.6, 10 mM 2,4,6-tri(2-pyridyl)-s-triazine (TPTZ) solution in 40 mM HCl, and 20 mM FeCl_3_·6H_2_O solution. Plant extracts or standard methanolic Trolox solutions (150 μL) were incubated at 37 °C with 2 mL of the FRAP solution (prepared by mixing 25 mL acetate buffer, 5 mL TPTZ solution, and 10 mL FeCl_3_·6H_2_O solution) for 30 min in the dark. Absorbance of the blue ferrous tripyridyltriazine complex formed was then read at 593 nm. Quantification was performed using a standard calibration curve of antioxidant Trolox (from 0.2 to 2.5 μmol/mL, *R*^2^: 0.995). Samples were analyzed in triplicate and results are expressed in μmol TE/100 grams fresh mass.

#### 3.5.2. Superoxide Anion Scavenging Activity

The enzyme xanthine oxidase is able to generate superoxide anion radical (O_2_^−^) “*in vivo*” by oxidation of reduced products from intracellular ATP metabolism. The superoxide anion generated in this reaction sequence reduces the nitroblue tetrazolium dye (NBT), leading to a chromophore with a maximum of absorption at 560 nm. Superoxide anion scavengers reduce the speed of generation of the chromophore. The superoxide anion scavenging activities of isolated compounds and fractions were measured spectrophotometrically in a microplate reader as reported previously [12]. All pear extracts were evaluated at 100 μg/mL. Values are presented as mean ± standard deviation of three determinations.

#### 3.5.3. Polyphenol and Flavonoids Contents

The total polyphenolic contents (TPC) of pear fruits were determined by the Folin-Ciocalteau method [13,14,34] with some modifications. An aliquot of each processed SPE extract (200 μL, approx. 2 mg/mL) was added to the Folin-Ciocalteau reagent (2 mL, 1:10 *v*/*v* in purified water) and after 5 min of reaction at room temperature (25 °C), 2 mL of a 100 g/L solution of Na_2_CO_3_ was added. Sixty minutes later the absorbance was measured at 710 nm. The calibration curve was performed with gallic acid (concentrations ranging from 16 to 500 μg/mL, *R*^2^ = 0.999) and the results were expressed as mg gallic acid equivalents/100 g fresh mass. Determination of total flavonoid content (TFC) of the methanolic extracts was performed as reported previously [34] using the AlCl_3_ colorimetric method. Quantification was expressed by reporting the absorbance in the calibration graph of quercetin, which was used as a standard (from 0.1 to 65.0 μg/mL, *R*^2^ = 0.994). Results are expressed as mg quercetin equivalents/g fresh weight. All spectrometric measurements were performed using a Unico 2800 UV-vis spectrophotometer (Unico Instruments, Co, Ltd., Shanghai, China).

### 3.6. Statistical Analysis

The statistical analysis was carried out using the originPro 9.0 software packages (Originlab Corporation, Northampton, MA, USA). The determination was repeated at least three times for each sample solution. Analysis of variance was performed using ANOVA. Significant differences between means were determined by Tukey comparison test (*p* values < 0.05 were regarded as significant).

## 4. Conclusions

The peel of the small Easter Pear peel presented the higher antioxidant activity measured by the bleaching of the radical DPPH, the ferric reducing antioxidant power (FRAP) and the superoxide anion scavenging activity (SA) assays (8.61 ± 0.65 μg/mL, 712.63 ± 12.12 μmol TE/100 g FW, and 82.89% ± 2.52% at 100 μg/mL, respectively). Apparently, this activity can be related to the presence of several phenolic compounds. It also showed higher flavonoid and total phenolic content than in the other tested varieties. Furthermore, the UHPLC fingerprints obtained pointed out that the methodology developed is appropriate for rapid analysis and identification of several phenolics in extracts from native pear fruits and could be used for other edible South American fruits. The fast UHPLC separation employed a linear gradient of 15 min with a solvent system of 0.1% aqueous formic acid (solvent A) and acetonitrile 0.1% formic acid (solvent B) with a flow rate of 1.0 mL·min^−1^. Twenty five compounds including one 3,5,4′-trihydroxy-7-methoxyflavone (peak 22), three chlorogenic acid isomers (peaks 3, 4, 8) and their dimers (peaks 17 and 19), six procyanidins (peaks 5, 6, 7, 9, 14 and 23) one *p*-coumaroylmalate (peak 15), one coumaroylquinic acid (peak 24), one caffeoyl glycoside (peak 1), four isorhamnetin derivatives (peaks 13, 16, 20 and 21) two quercetin derivatives (peaks 11 and 12), one feruloylquinic acid (peak 10) and one kaempferol derivative (peak 25) were identified in a native small pear from an oasis of the II region of Chile using PDA and OT-ESI-MS for the first time. Significant differences in the total phenolic content and antioxidant activity were found between these native pears and two other pears commercialized in Chile. The small pears are good candidates for industrial crop production and their peels have potential use to produce nutraceuticals, according to the high antioxidant activity found and the presence of dietary phenolic compounds.
